# Development of retrieval-augmented generation–based large language model for drug-induced liver injury using Livertox data

**DOI:** 10.1097/HC9.0000000000000895

**Published:** 2026-02-12

**Authors:** Ashwin Rao, George Cholankeril, Avegail Flores, Gagan Sood, Terrence E. White, Fasiha Kanwal, Hashem B. El-Serag

**Affiliations:** 1 Section of Gastroenterology and Hepatology, Department of Medicine, Baylor College of Medicine, Houston, Texas, USA; 2 D. Bradley McWilliams School of Bioinformatics, University of Texas-Health at Houston, Houston, Texas, USA

**Keywords:** artificial intelligence, clinical decision support, DILI, hepatotoxicity

## Abstract

**Background:**

Idiosyncratic DILI is a complex clinical challenge requiring timely and accurate decision support. LiverTox, curated by the National Institute of Health (NIH), offers a comprehensive DILI evidence base, but its encyclopedia-like format hinders point-of-care use. Health care providers increasingly use general large language models (LLMs) for clinical care, raising safety concerns due to LLM hallucinations or misinformation. We hypothesize that retrieval-augmented generation (RAG) integration—grounding LLM responses in LiverTox content—would enable accurate DILI decision support.

**Methods:**

We processed 1343 LiverTox drug monographs into 8759 indexed segments using BioBERT embeddings. We developed a RAG pipeline that employs drug-specific prioritization, section-aware weighting, and semantic search to retrieve the most relevant content per query. Twenty-five DILI questions were evaluated across 6 models: 4 RAG-LLMs: Mistral-7B, Claude-3-Haiku, Claude-3-Opus, and GPT-4o, and 2 non-RAG GPT-4o variants (unconstrained; soft constrained with a prompt to reference LiverTox). Three hepatologists, blinded to the model, evaluated responses for accuracy, completeness, and conciseness using 5-point Likert scales. Analyses included pairwise comparisons and effect size estimation.

**Results:**

One hundred fifty model responses were evaluated with good inter-rater reliability. GPT-4o (RAG) achieved the highest overall scores (4.47±0.10). RAG-LLMs outperformed non-RAG GPT-4o variants in accuracy (*p*<0.001) and completeness (*p*<0.01). Moderate to large effect sizes in accuracy (d=0.778) and completeness (d=0.526) were noted with RAG. No hallucinations were observed in RAG-LLM outputs, while both non-RAG GPT-4o variants produced several hallucinated responses. There were no significant differences in scoring or hallucinated response rate between the 2 non-RAG variants.

**Conclusions:**

We developed an RAG-LLM integrated with LiverTox for evidence-based DILI management. RAG-LLM systems outperformed non-RAG variants and produced responses without observed hallucinations in this evaluation. Our LiverTox RAG-LLM enables reliable answers to drug hepatotoxicity questions at the point of care.

## INTRODUCTION

Idiosyncratic DILI refers to drug-induced liver damage or dysfunction that occurs independent of dose and duration of administration.^1^ Population-based studies estimate the incidence of DILI to be 14–19 cases per 100,000 people,^2^^,^^3^ and its severity can range from transient laboratory abnormalities to acute liver failure leading to hospitalization, need for emergent liver transplantation, or death.

Outcomes in idiosyncratic DILI remain poor in comparison to other etiologies of liver injury, emphasizing the importance of early identification of the causative drug, cessation, and appropriate treatment.^4^ However, identification of causative agents remains challenging due to polypharmacy and a lack of objective diagnostic markers. To assist clinicians, the National Institute of Health (NIH) developed LiverTox to provide evidence-based hepatotoxicity profiles of over 1300 medications.^5^ LiverTox remains the authoritative guideline-endorsed resource for clinicians for the management of DILI.^6^ However, it presents significant usability challenges at the point of care due to its traditional search interface and dictionary-like structure, which requires extensive manual filtering and increased cognitive load for users to retrieve clinically relevant insights.

Large language models (LLMs) offer conversational interfaces for natural language queries that may improve clinical information retrieval, summarization, and decision support for users.^7^ Health care institutions are increasingly deploying HIPAA-compliant versions of general-purpose LLM models (eg, ChatGPT or Copilot) for clinicians to query during clinical care^8^; however, LLM responses are susceptible to “hallucinations”—fabricated or factually incorrect answers, or misinformation retrieved from nonevidence-based sources.^9^ These are unacceptable risks in clinical settings. Retrieval-augmented generation (RAG) grounds LLM responses in verified information sources and has been shown to reduce hallucination rates.^10^ Recent meta-analyses demonstrate that RAG implementation leads to significantly improved performance in LLM-based clinical decision-making and medical question-answering in specialties including gastroenterology, hepatology, internal medicine, oncology, neurology, and ophthalmology.^11^ When integrated with evidence-based sources, LLMs can transform static knowledge repositories into dynamic decision support tools, as demonstrated by LiVersa—a liver-specific RAG-LLM clinician chatbot interface developed with American Association for the Study of Liver Diseases (AASLD) guidelines.^12^ To address the usability barriers of LiverTox while mitigating the challenges of hallucination and possible misinformation provided by commercial LLMs, we developed and evaluated a RAG-LLM system based on LiverTox for DILI management. We hypothesized that a RAG-LLM system, in comparison to non-RAG-based models, would deliver accurate, real-time DILI guidance without hallucinations (Figure 1).

**FIGURE 1:**
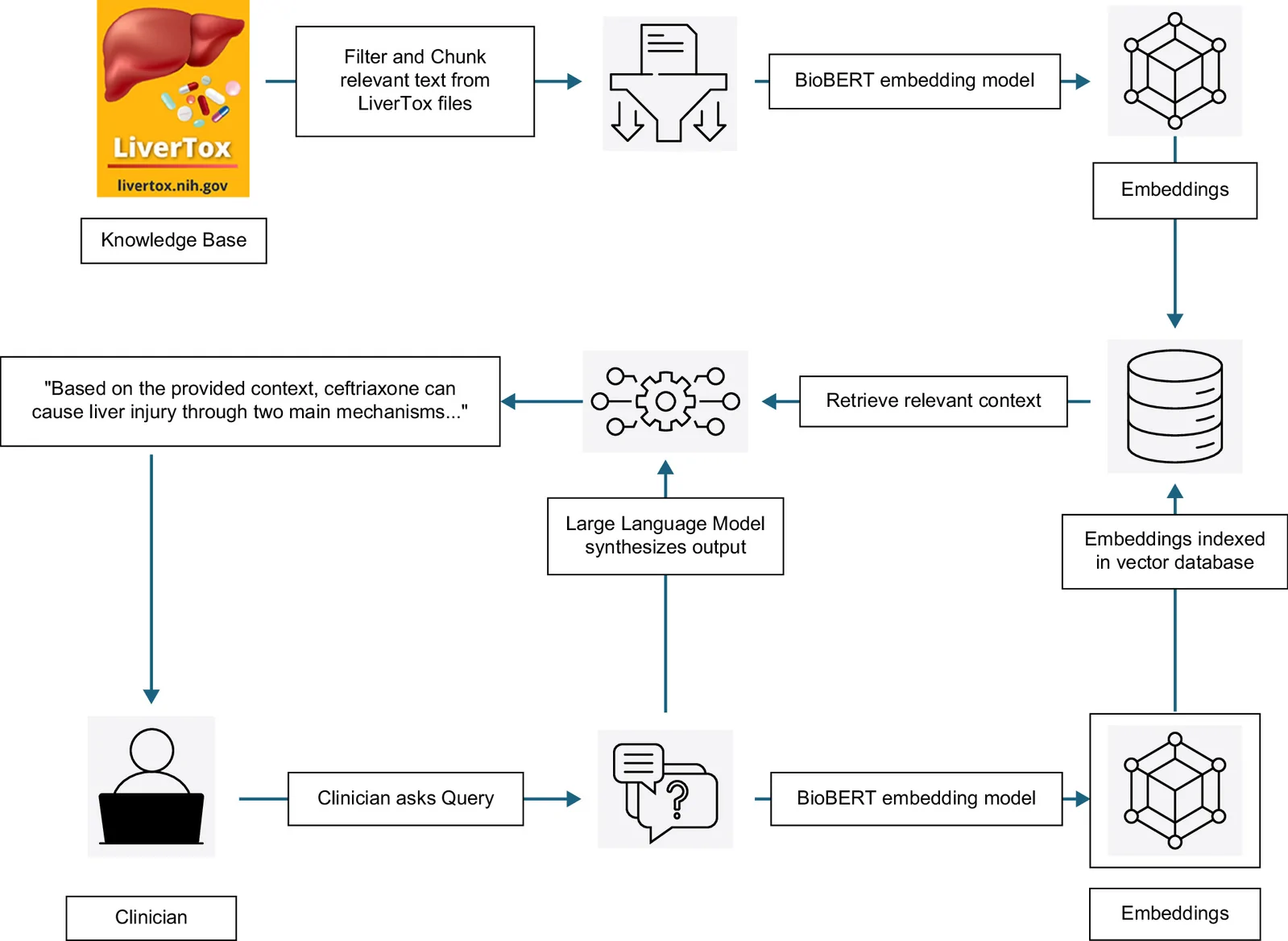
LiverTox RAG-LLM pipeline. The system extracts and filters clinically relevant sections from LiverTox documents, segments them into chunks, and embeds them using a BioBERT model. Embeddings are indexed in a vector database for efficient retrieval. When a clinician submits a query, relevant context is retrieved and passed to an LLM, which synthesizes a response grounded in the retrieved context.

## METHODS

Informed consent and institutional review board approval were not required due to the use of public data.

### Processing the LiverTox knowledge base

We systematically processed 1343 LiverTox drug files into 8759 semantically meaningful segments, entailing the following steps. Drug monographs in NXML format were parsed to extract clinically relevant sections: Introduction, Background, Hepatotoxicity, Mechanism of Injury, Outcome and Management, Product Information, and Case Reports. Text within each section was chunked into segments of ~1500 characters while preserving paragraph boundaries. Each chunk retained drug and section metadata and document identifiers.

### Generating embeddings

To enable the system to interpret medical language and retrieve relevant content, text segments were converted into numerical representations called embeddings, which capture their underlying meaning. We generated these embeddings with BioBERT, a language model trained on biomedical literature and optimized to capture clinically relevant concepts. These embeddings were then organized using Facebook AI Similarity Search, a specialized search tool that efficiently compares these representations to identify and retrieve the most relevant sections from thousands of LiverTox documents.

### Developing the RAG pipeline

We designed a retrieval system combining drug-specific filtering, section-aware weighting, and semantic similarity search. When a drug name was detected in the query, the system restricted retrieval to chunks from that specific drug’s monograph. Retrieved chunks were then ranked using a weighted scoring function that combined: (1) cosine similarity between query and chunk embeddings, (2) drug relevance boosting, and (3) section-aware prioritization. The top 5 highest-scoring chunks were provided as context for RAG-LLM systems to formulate responses to user queries. RAG-LLM responses included citations referencing the specific LiverTox monograph sections used to generate each answer and ensure traceability of all referenced material.

### LLM selection

We evaluated 6 models varying in size (7B-1.8T parameters), context length (32K-200K tokens), availability (open/closed-source), training cutoff, tokenizer, and retrieval approach (RAG/non-RAG) (Table 1). RAG-LLM systems used GPT-4o, Claude-3-Opus, Claude-3-Haiku, and Mistral-7-B-Instruct-v0.1. For non-RAG comparisons, we selected GPT-4o, given its position as one of the most widely used and publicly available LLMs. There were 2 non-RAG comparisons: GPT-4o “without constraints” (no specific prompt instructions to reference LiverTox when answering questions) and GPT-4o with “soft constraints” (prompt instructions to only reference LiverTox when answering questions). In contrast, RAG (“hard constraints”) methods restricted the model’s accessible context to only those LiverTox passages retrieved through our retrieval pipeline, preventing exposure to any external data sources (Supplementary Table 1, http://links.lww.com/HC9/C260). All 6 models were otherwise given a standardized prompt and set to a temperature of 0—a parameter that controls the variability of generated text—to facilitate near-deterministic output and maximize reproducibility. All models were accessed via application programming interface, except for the open-source Mistral-7B-Instruct v0.1, which was deployed locally.

**TABLE 1:** Model specifications and characteristics

Model	Company	Parameters	Context window (tokens)	Estimated date of training cutoff	Tokenizer	Access source	Type
GPT-4o (RAG)	OpenAI	~1.8 trillion^a^	128,000	October 2023	Tiktoken	API	RAG
Claude Opus (RAG)	Anthropic	~1 trillion^a^	200,000	August 2023	Not disclosed	API	RAG
Claude Haiku (RAG)	Anthropic	~100 billion^a^	200,000	August 2023	Not disclosed	API	RAG
MISTRAL-7B (RAG)	Mistral AI	7 billion	32,000	December 2023	Sentence Piece	Local	RAG
GPT-4o (No constraints)	OpenAI	~1.8 trillion	128,000	October 2023	Tiktoken	API	Non-RAG
GPT-4o (Soft constraints)	OpenAI	~1.8 trillion	128,000	October 2023	Tiktoken	API	Non-RAG

*Note:* Specifications of evaluated large language models. RAG models use retrieval-augmented generation with LiverTox database content as detailed in Figure 1.

^a^ Estimated parameters based on publicly available information

Abbreviations: API, Application Programming Interface; RAG, retrieval-augmented generation.

### Evaluation protocol

We developed a standardized evaluation instrument comprising 25 DILI-focused questions through iterative consensus by 3 board-certified transplant hepatologists. Questions were systematically selected to assess LiverTox retrieval and clinical relevance across essential decision support topics: hepatoxicity patterns, mechanisms of injury, clinical features, and management (Supplementary Table 2, http://links.lww.com/HC9/C260). These same three hepatologists, instructed to consult LiverTox as their reference standard and blinded to model identity, independently evaluated randomized model responses on a 5-point Likert scale (1=poor, 2=marginal, 3=acceptable, 4=very good, 5=excellent) in 3 domains: accuracy (factual correctness), completeness (comprehensiveness), and conciseness (clarity without excess). Hallucinations were identified by cross-verifying each model response against the LiverTox reference standard. Statements not present in LiverTox, contradictory to LiverTox, or fabricating associations were classified as hallucinated responses.

### Statistical analysis

Inter-rater reliability in accuracy, completeness, and conciseness domains was assessed using intraclass correlation coefficients based on a two-way mixed-effects model for single measures (intraclass correlation coefficient [2,1]). Due to non-normal score distributions (Shapiro-Wilk *p* <0.001 for all domains), model performance was compared using Kruskal-Wallis tests, followed by pairwise Mann-Whitney *U* tests with Bonferroni correction (α=0.0033). A post hoc analysis to evaluate whether variability in document length (chunk count per drug) influenced model accuracy. Effect sizes were calculated for domain differences between RAG and non-RAG models using Cohen *d* (standardized mean difference, where values of ~0.2, 0.5, and 0.8 typically indicated small, medium, and large effects, respectively).^13^ A priori, we defined a ≥0.5-point difference on the 5-point Likert scale as clinically meaningful, consistent with previous psychometric evidence that half a SD represents a minimally important difference across rating instruments.^14^

Code generation and statistical analysis were implemented in Python 3.11.

## RESULTS

A total of 150 model responses (6 models for 25 questions) were evaluated by 3 expert hepatologists; inter-rater reliability was good across all domains (overall, 0.693; accuracy, 0.68; completeness, 0.62; conciseness, 0.78). Post hoc analysis demonstrated no association between chunk count and model accuracy (Pearson *r*=–0.04, *p*=0.87; Spearman *p*=–0.06 and 0.80).

Across all RAG-based systems, relevant LiverTox drug entries and filtered content sections were successfully retrieved for 100% (25/25) DILI queries. GPT-4o (RAG) achieved the highest mean ratings overall (mean±SE, 4.47±0.10) with “very good” to “excellent” scores across each domain (accuracy, 4.58±0.09; completeness, 4.26±0.12; conciseness, 4.58±0.10). Claude Opus (RAG) received strong overall ratings with high accuracy and completeness scores, though it was less concise. Claude Haiku (RAG) scored slightly lower in accuracy and completeness, and the lowest of all models in conciseness. Mistral-7B (RAG) demonstrated intermediate performance.

Both non-RAG models, GPT-4o without constraints and GPT-4o with soft constraints, received the lowest overall scores, particularly with “average” scores in domains of accuracy and completeness of response (Table 2, Figure 2). No statistically significant differences were observed between GPT-4o (soft constraints) and GPT-4o (no constraints) across any domain: accuracy (*p*=0.103), completeness (*p*=0.514), or conciseness (*p*=0.865).

**TABLE 2:** Model performance summary statistics

Model	Accuracy (mean, SE)	Completeness (mean, SE)	Conciseness (mean, SE)	Overall (mean, SE)
GPT-4o (RAG)	4.58±0.09	4.26±0.12	4.58±0.10	4.47±0.10
Claude Opus (RAG)	4.40±0.14	4.10±0.15	4.02±0.13	4.17±0.14
Claude Haiku (RAG)	4.30±0.10	3.98±0.13	3.08±0.18	3.79±0.14
MISTRAL-7B (RAG)	3.78±0.16	3.56±0.18	3.94±0.14	3.76±0.16
GPT-4o (No constraints)	3.64±0.14	3.50±0.13	3.86±0.16	3.67±0.15
GPT-4o (Soft constraints)	3.24±0.2	3.36±0.17	3.82±0.17	3.47±0.18

*Note:* Performance metrics across 3 evaluation domains: accuracy, completeness, and conciseness. Values represent mean scores, on a 1–5 Likert scale (1=poor, 2=marginal, 3=acceptable, 4=very good, 5=excellent), with SEs. GPT-4o (RAG) achieved the highest scores in each domain, with ratings between “very good” and “excellent.” Non-RAG models received the lowest scores, in the “acceptable” range for accuracy and completeness.

**FIGURE 2:**
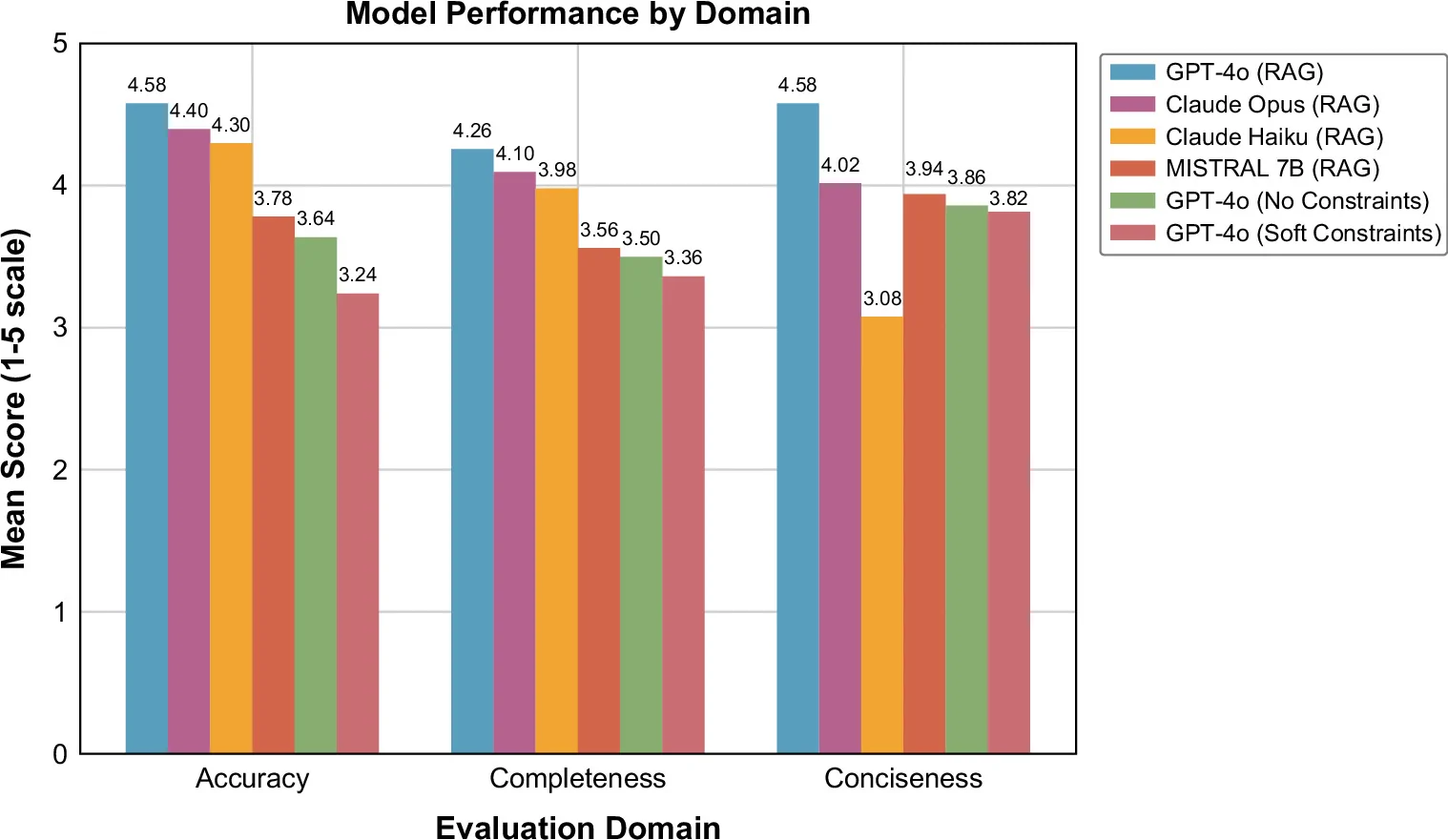
Model performance by domain. Comparative performance of mean scores of all models across each of the 3 evaluation domains. GPT-4o (RAG) achieved the highest scores in each domain. Non-RAG models received the lowest scores in accuracy and completeness, while the Claude Haiku (RAG) model received lower scores in conciseness. Abbreviation: RAG, retrieval-augmented generation.

Pairwise statistical testing comparing each model’s performance, defined as mean domain scores in accuracy, completeness, and conciseness showed GPT-4o (RAG) significantly outperformed all other models in accuracy, except for Claude Opus (RAG; *p*=0.15) and Haiku (RAG; *p*=0.08), where the high performance did not reach significance (Figure 3, Supplemental Table S3, http://links.lww.com/HC9/C260). In terms of completeness, GPT-4o (RAG) was again significantly superior to all other implementations except against Claude Opus (RAG; *p*=0.21) and Haiku (RAG; *p*=0.11), where the high performance did not reach significance. When assessing conciseness, GPT-4o (RAG) significantly outperformed all other models. Each of the above comparisons with GPT-4o (RAG), except Claude Opus and Haiku in accuracy and completeness, met the criteria for a clinically meaningful difference.

**FIGURE 3:**
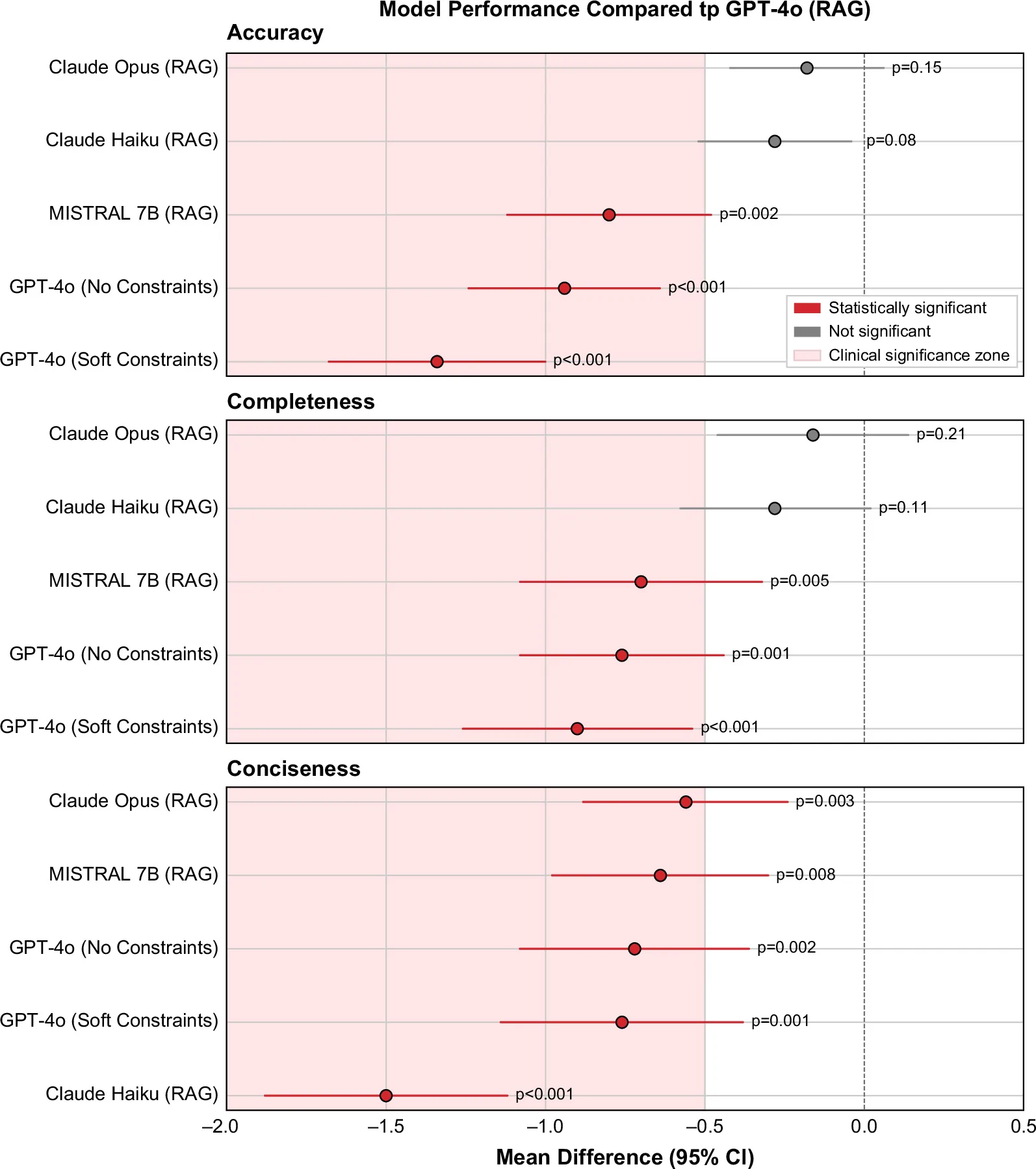
Forest plot. Forest plot with 95% CIs comparing each model to GPT-4o (RAG) baseline. Red shading indicates zone of clinical significance (>0.5-point difference in Likert score). All models received lower scores in each domain compared to GPT-4o. Lower scores in accuracy and completeness for Claude Opus and Haiku did not reach statistical significance.

RAG-LLM systems received significantly higher scores than non-RAG models in both accuracy (*p*<0.001) and completeness (*p*<0.01) (Figure 4A). Conciseness did not differ significantly between the 2 groups. Corresponding effect sizes for RAG implementation showed a medium to large effect in accuracy and completeness (d=0.778, d=0.526, respectively) (Figure 4B).

**FIGURE 4:**
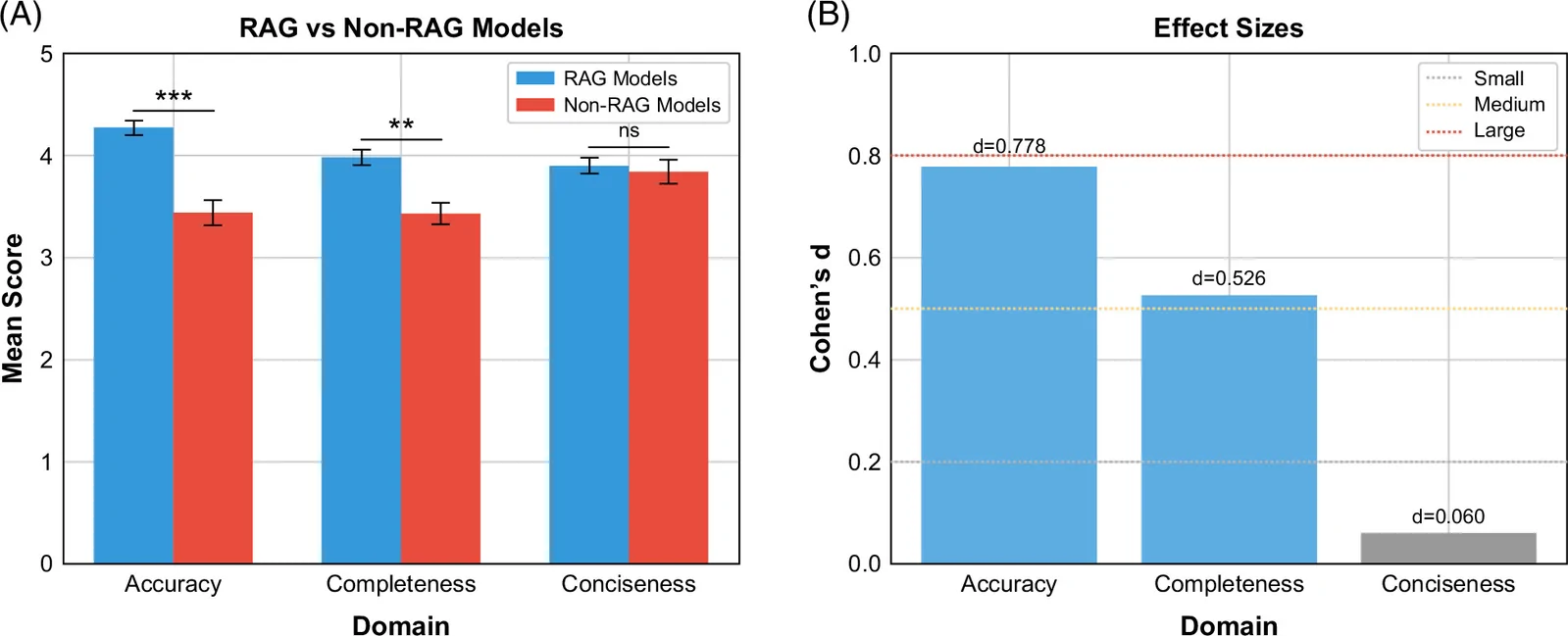
Comparison of RAG versus non-RAG models. Comparison of RAG versus non-RAG models. (A) Mean scores with standard errors and statistical significance (*p<0.05, ** *p*<0.01, ***p<0.001). RAG models significantly outperformed non-RAG models in accuracy and completeness. (B) Cohen d effect sizes approach large effects for accuracy and show medium effects for completeness, indicating meaningful improvements in each domain with RAG implementation.

None of the 4 RAG models provided a hallucinated response, with every answer traceable to LiverTox content; sample responses from GPT-4o (RAG) are shown in Table 3. Both non-RAG implementations provided several hallucinated responses, ranging from incorrect LiverTox likelihood scores to fabricated mechanisms of injury, timing of DILI onset, and drug associations with the development of HCC or cirrhosis (Table 3).

**TABLE 3:** Representative responses generated by GPT-4o models with and without retrieval-augmented generation

Drug	GPT-4o RAG-LLM	GPT-4o (non-RAG)	Error type
Phenytoin	“The **likelihood score for phenytoin causing liver injury is A** (well-known cause of clinically apparent liver injury).” <Phenytoin: Hepatotoxicity, Phenytoin: Mechanism of Injury, Phenytoin: Outcome and Management, Phenytoin: Case 1. Acute immunoallergic hepatitis 3 weeks after starting phenytoin, Phenytoin: Background>	No constraints: “Phenytoin has a **likelihood score of D** for causing liver injury, indicating a probable but rare cause”	Incorrect likelihood score; correct score of A.
Ketoconazole	“…**The liver injury is clearly idiosyncratic**, and ketoconazole is a potent inhibitor of human CYP 3A4, which can alter the serum levels of many drugs metabolized via the P450 system…” <Ketoconazole: Mechanism of Injury, Ketoconazole: Outcome and Management, Ketoconazole: Case 4. Acute liver failure due to ketoconazole, Ketoconazole: Case 2. Ketoconazole-induced acute, self-limited hepatitis. Ketoconazole: Case 1. Acute self-limited serum enzyme elevations during ketoconazole therapy>	No constraints: “The risk of liver **injury is dose-dependent** and can vary among individuals”	Injury idiosyncratic, not dose-dependent
Duloxetine	“The **likelihood score of injury from duloxetine is B** (likely cause of clinically apparent liver injury). The **onset of injury is usually within 1 to 6 months**.” <Duloxetine: Mechanism of Injury, Duloxetine: Introduction, Duloxetine: Hepatotoxicity, Duloxetine: Outcome and Management, Duloxetine: Background>	No constraints: “The likelihood score of drug-induced liver injury from duloxetine is **rated as a 'C'** on the likelihood scale…the **onset of injury typically occurs within 1 to 3 months of starting the medication.”**	Incorrect likelihood score; correct score of B. Incorrect onset timing (1–6 mo)
Amiodarone	“Amiodarone can cause two distinct types of liver injury. When given orally for long-term therapy, it can lead to chronic liver injury that is prolonged and resolves slowly over months. **This chronic injury can result in permanent damage, such as hepatomegaly and cirrhosis**.” <Amiodarone: Outcome and Management, Amiodarone: Hepatotoxicity, Amiodarone: Case 2. Cirrhosis with decompensation but ultimate recovery from long-term therapy with amiodarone, Amiodarone: Mechanism of Injury, Amiodarone: Case 1. Cirrhosis and end-stage liver disease from long-term therapy with amiodarone>	No constraints: “Oral administration of amiodarone can lead to chronic liver injury, which often presents as steatosis (fatty liver), hepatitis, cirrhosis, **or even hepatocellular carcinoma with long-term use.”**	Fabricated association of medication use with the development of HCC
Nitrofurantoin	“The severity of nitrofurantoin-induced liver injury can **range from mild elevations in serum aminotransferase levels to hepatitis with jaundice, and in severe cases, fulminant liver failure and death.** Complete recovery is expected after stopping the drug, but recovery may be slow, taking 2 to 6 months. **In rare cases, evidence of chronic liver injury may persis**t.” <Nitrofurantoin: Outcome and Management, Nitrofurantoin: Case 1. Chronic hepatitis with hepatocellular pattern of serum enzyme elevations due to nitrofurantoin, Nitrofurantoin: Hepatotoxicity, Nitrofurantoin: Mechanism of Injury, Nitrofurantoin: Case 4. Acute liver failure due to nitrofurantoin>	No constraints: “In some instances, particularly with prolonged exposure, serious outcomes such as chronic injury, fulminant liver failure, **or cirrhosis may occur”**	Fabricated association of prolonged exposure with the development of cirrhosis.
Phenytoin		Soft constraints: “Phenytoin has a **likelihood score of C** for causing liver injury according to the LiverTox database.”	Incorrect likelihood score; correct score of A.
Duloxetine		Soft constraints: “According to the LiverTox database, Duloxetine has a **likelihood score of C** for causing drug-induced liver injury. The **onset of liver injury typically occurs within 1 to 8 weeks of starting the medication.”**	Incorrect likelihood score; correct score of B. Incorrect onset timing (1–6 mo)

*Note:* Examples of correct responses from GPT-4o RAG-LLM and hallucinated responses generated by both GPT-4o models (no constraint and soft constraint). RAG-LLM responses were fully traceable to LiverTox Source sections used in response generation.

## DISCUSSION

We developed and evaluated a RAG-LLM system based on LiverTox for DILI management, demonstrating that RAG-LLM systems outperform non-RAG models in effectively synthesizing information to answer DILI-related clinical queries.

GPT-4o (RAG) achieved the highest overall performance, and RAG-LLM systems significantly outperformed non-RAG models in response accuracy and completeness of answer. In addition, moderate to large effect size differences in accuracy and completeness of answer domains suggest that the implementation of RAG leads to clinically meaningful improvements in the veracity and reliability of DILI-specific LLM answers. These results are in line with several studies of RAG-LLM systems across diverse medical specialties, with pooled data reinforcing that using RAG to ground LLM responses in trusted, domain-specific sources improves performance.^11^^,^^15^^–^^17^

In addition to statistical superiority, RAG-LLM models demonstrated a clear safety advantage. No hallucinated responses were observed across any RAG-generated outputs, whereas both non-RAG models produced multiple hallucinated responses. Hallucinated responses included incorrect risk classifications (phenytoin, duloxetine), fabricated mechanisms (ketoconazole-induced DILI dose-dependent), and incorrect links between hepatotoxic agents with the development of HCC and cirrhosis (amiodarone and nitrofurantoin, respectively). If deployed for point-of-care use, such responses could lead to incorrect or delayed identification of the putative agent, drug dose reduction rather than appropriate drug withdrawal, or patient harm due to false associations with cancer and advanced liver disease. Given that users often overestimate the capabilities and factual accuracy of LLMs,^18^ mitigation strategies that eliminate hallucinations, rather than relying on clinician judgment to detect errors in responses, are vital for accurate and trustworthy AI-enhanced decision support.

Increasing model size and reasoning capabilities have been suggested as factors that may reduce hallucinated response rates.^19^ However, our findings suggest that these factors alone are insufficient without RAG. For instance, Mistral-7B, a 7-billion parameter model with limited reasoning capabilities, produced no hallucinated responses when paired with RAG. Conversely, GPT-4o—despite its advanced reasoning capabilities and estimated 1.8 trillion parameter size—generated multiple hallucinations in non-RAG implementations. Notably, recent OpenAI safety card data show that internal testing of newer, larger models with more advanced reasoning capabilities, such as GPT-o3, demonstrates increased hallucinated response rates.

Prompt engineering has also been cited as a mitigation strategy for LLM hallucinations,^20^^,^^21^ such as a prompt instruction to limit responses to solely LiverTox content. However, our results demonstrate that soft-constrained prompting—instructing the model to only reference LiverTox—showed no significant advantage over an unconstrained prompt, with comparable domain scores and similar rates of hallucinated responses. Notably, the soft-constrained GPT-4o model frequently prefaced answers with “According to the LiverTox database,” yet still produced incorrect responses, for instance, misreporting Duloxetine’s likelihood score and time to onset. This mirrors prior findings that LLMs may generate fabricated or misattributed citations that appear superficially credible^19^ and underscores a broader concern in model use for clinical decision support: hallucinated content, when framed confidently, may engender misplaced clinician trust in responses. In contrast, a RAG-based system enhances trustworthiness by ensuring traceability through the retrieval and citation of specific LiverTox content used to formulate each response.

Our study has limitations. We evaluated model performance using a standardized question set, which captures the full complexity or ambiguity of real-world scenarios as compared to clinical cases or patient-specific data. However, most prior LLM evaluations have relied on multiple-choice question formats, which offer even lower fidelity; our free-text clinical queries allow for a more nuanced assessment and were sufficient to detect meaningful differences in model performance. Our evaluation focused on well-established hepatotoxic agents with extensive LiverTox documentation to ensure reliable comparison with a comprehensive reference standard. Future studies should assess newer or less complete entries, where limited source data may restrict RAG outputs and training cutoffs may increase hallucination risk in non-RAG models. Similarly, we evaluated generic drug names aligned with LiverTox entries. Brand name variations were not assessed, though this limitation will be addressed through integration with standardized drug name databases for automatic normalization. LiverTox entries vary in length and completeness, with some drugs containing extensive case reports and others having shorter documentation, which could influence retrieval and model accuracy. However, our section-aware weighting prioritized clinically salient content, and post hoc analysis indicated that variability in document length did not affect model performance. While expert hepatologists rated the quality of model responses, we did not assess the downstream clinical impact of LLM-generated outputs on decision-making or patient outcomes. Though additional prompt engineering techniques may have improved non-RAG performance, we prioritized standardized prompts to reflect real-world use, where extensive prompt optimization is unlikely. Finally, our evaluation focused exclusively on LiverTox data, which, while authoritative, does not encompass all sources of hepatotoxicity data or account for emerging literature. However, we felt it was critical to anchor model outputs in a trusted, verifiable source of truth before expanding to less curated knowledge bases.

A DILI RAG-LLM system may enhance the accessibility of LiverTox for frontline clinicians, addressing a key barrier in translating a rich knowledge base into actionable bedside clinical support. With further development, integration into the electronic health record could allow the system to process clinician queries alongside patient-specific history and laboratory values, and automatically generate structured causality assessments using established frameworks, such as the Roussel Uclaf Causality Assessment Method. Prior to public or clinical deployment, future work will focus on (1) supervised fine-tuning on expert-authored question-answer pairs to align responses with clinical decision making, (2) stepwise testing on real patient cases that incorporate clinical histories, laboratory data, and multiple implicated drugs, (3) integration within secure, HIPAA-compliant systems, and (4) prospective evaluation in clinical workflows to determine usability and impact on patient outcomes.

In this study, we developed and rigorously evaluated the first RAG-LLM system grounded in the LiverTox database for DILI. We show that the implementation of RAG eliminated hallucinated responses while significantly improving performance in knowledge retrieval and clinical question-answering. RAG-based LLMs could be similarly developed to improve access and usability across complex, expert-curated GI-based knowledge repositories—from polyp surveillance guidelines to viral hepatitis management. As clinical use of LLMs continues to expand, grounding responses in trusted, domain-specific sources will be essential to developing safe and effective decision-support tools.

## Supplementary Material
