# Supplementary material for: Development of retrieval-augmented generation–based large language model for drug-induced liver injury using Livertox data
Source: Hepatol Commun. 2026 Feb 12;10(3):e00895. doi: 10.1097/HC9.0000000000000895 (PMC13671588; doi:10.1097/HC9.0000000000000895)
Supplement: Supplementary file 1 [file hc9-10-e00895-s001.docx]

**Supplementary Table 1. Question and Answer Set.** A standardized DILI question set of twenty-five clinically relevant queries on hepatoxicity patterns, mechanisms of injury, clinical features, and management was developed and used to evaluate each model.

**Supplementary Table 2. Pairwise Statistical Comparisons by Model.** Pairwise comparisons of all models against GPT-4o (RAG) as the reference standard. Bolded P-values < 0.0033 indicate statistical significance after Bonferroni correction. All models received lower scores in each domain compared to GPT-4o. Lower scores in accuracy and completeness for Claude Opus and Haiku did not reach statistical significance.

**Supplementary Table 3. Prompt Templates for Model Evaluation**. Exact prompt templates provided to all models in the non-RAG (no constraint), non-RAG (soft constraint), and RAG comparisons.

**Supplementary Table 1.**

Question

What kind of liver injury does Amiodarone cause? Is it different when given intravenously?

What are the main features of hepatotoxicity from Methotrexate?

Does Atorvastatin cause acute liver failure?

What is the likelihood score of injury from Isoniazid?

What is the mechanism of liver injury from Valproate?

What is the outcome and management of liver injury from Nitrofurantoin?

What is the typical onset of liver injury from Azathioprine?

Is liver injury from Diclofenac typically hepatocellular, cholestatic, or mixed?

What is the likelihood score for Phenytoin causing liver injury?

What is the proposed mechanism of liver injury for Atovaquone?

When is the onset of injury from Allopurinol?

How does Ketoconazole cause hepatotoxicity?

Is Minocycline associated with autoimmune liver injury?

What is the clinical presentation of liver injury from Atovaquone?

Does Valacyclovir cause liver injury?

What is the pattern of liver injury from Allopurinol?

What is the proposed mechanism for hepatotoxicity from Isoniazid?

Can Hydroxycut cause liver injury? If so, how?

What is the mechanism of liver injury from Methotrexate?

Can Ginkgo cause liver injury?

Can Moringa cause liver injury?

What liver biopsy findings are reported in cases of hepatotoxicity from Nitrofurantoin?

How does Ceftriaxone cause liver injury?

What is the likelihood score of injury from Halothane?

What is the likelihood score of injury from Duloxetine? When is the onset of injury?

**Supplementary Table 2.**

| **All Models compared to GPT-4o (RAG) Reference:** | | | | |
| --- | --- | --- | --- | --- |
| **Model** | **Domain** | **Mean Difference** | **95% CI** | **p-value** |
| MISTRAL 7B (RAG) | Accuracy | -0.80 | [-1.12, -0.48] | **0.002** |
| Claude Haiku (RAG) | Accuracy | -0.28 | [-0.52, -0.04] | 0.08 |
| Claude Opus (RAG) | Accuracy | -0.18 | [-0.42, 0.06] | 0.15 |
| GPT-4o (No Constraints) | Accuracy | -0.94 | [-1.24, -0.64] | **<0.001** |
| GPT-4o (Soft Constraints) | Accuracy | -1.34 | [-1.68, -1.00] | **<0.001** |
| MISTRAL 7B (RAG) | Completeness | -0.70 | [-1.08, -0.32] | **0.005** |
| Claude Haiku (RAG) | Completeness | -0.28 | [-0.58, 0.02] | 0.11 |
| Claude Opus (RAG) | Completeness | -0.16 | [-0.46, 0.14] | 0.21 |
| GPT-4o (No Constraints) | Completeness | -0.76 | [-1.08, -0.44] | **0.001** |
| GPT-4o (Soft Constraints) | Completeness | -0.90 | [-1.26, -0.54] | **<0.001** |
| MISTRAL 7B (RAG) | Conciseness | -0.64 | [-0.98, -0.30] | **0.008** |
| Claude Haiku (RAG) | Conciseness | -1.50 | [-1.88, -1.12] | **<0.001** |
| Claude Opus (RAG) | Conciseness | -0.56 | [-0.88, -0.24] | **0.003** |
| GPT-4o (No Constraints) | Conciseness | -0.72 | [-1.08, -0.36] | **0.002** |
| GPT-4o (Soft Constraints) | Conciseness | -0.76 | [-1.14, -0.38] | **0.001** |

| **Model** | **Prompt** |
| --- | --- |
| GPT-4o (No Constraints) | “Does atorvastatin cause acute liver failure? |
| GPT-4o (Soft Constraints) | “Answer the following question using only information from the NIH LiverTox database. If information is unavailable in LiverTox, state this explicitly. Be accurate, complete, and concise.  Question: Does atorvastatin cause acute liver failure?” |
| LLM (RAG) | “Based on the provided LiverTox content below, answer the clinical question using only the information in the context. If information is unavailable in LiverTox, state this explicitly. Be accurate, complete, and concise. Context: [RETRIEVED_CONTENT] Question: Does atorvastatin cause acute liver failure?” |

**Supplementary Table 3.**
